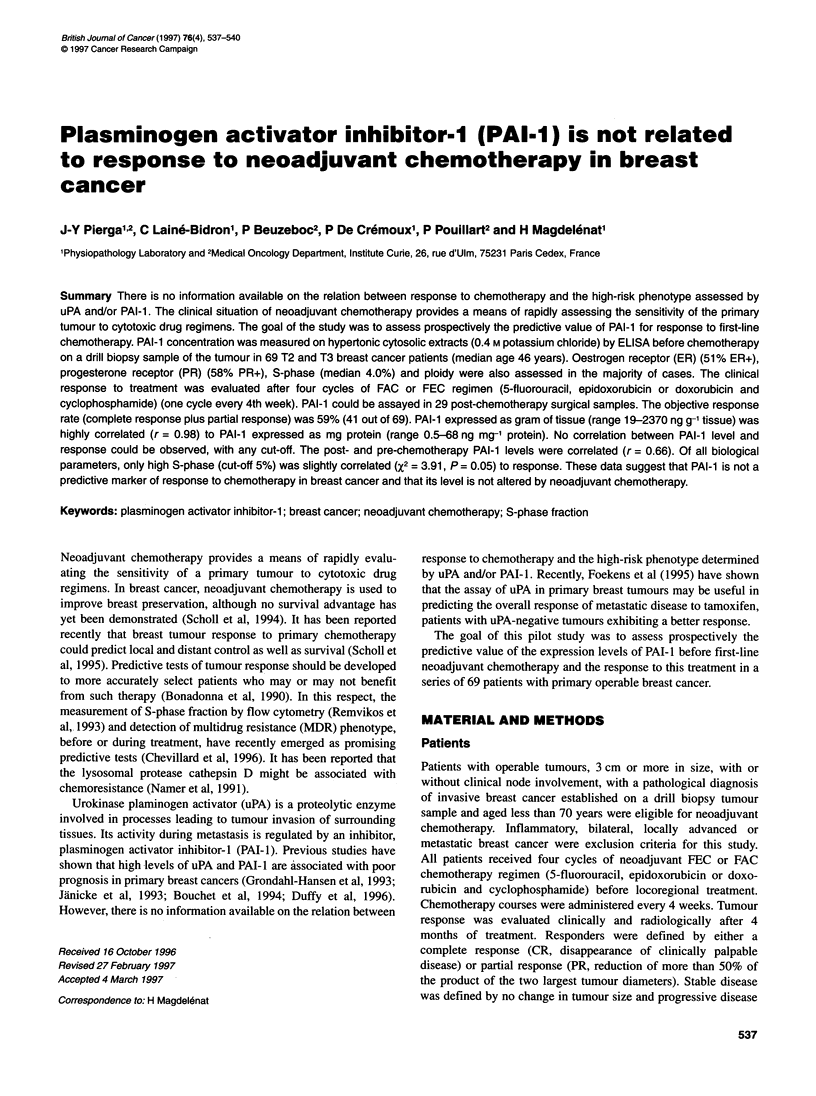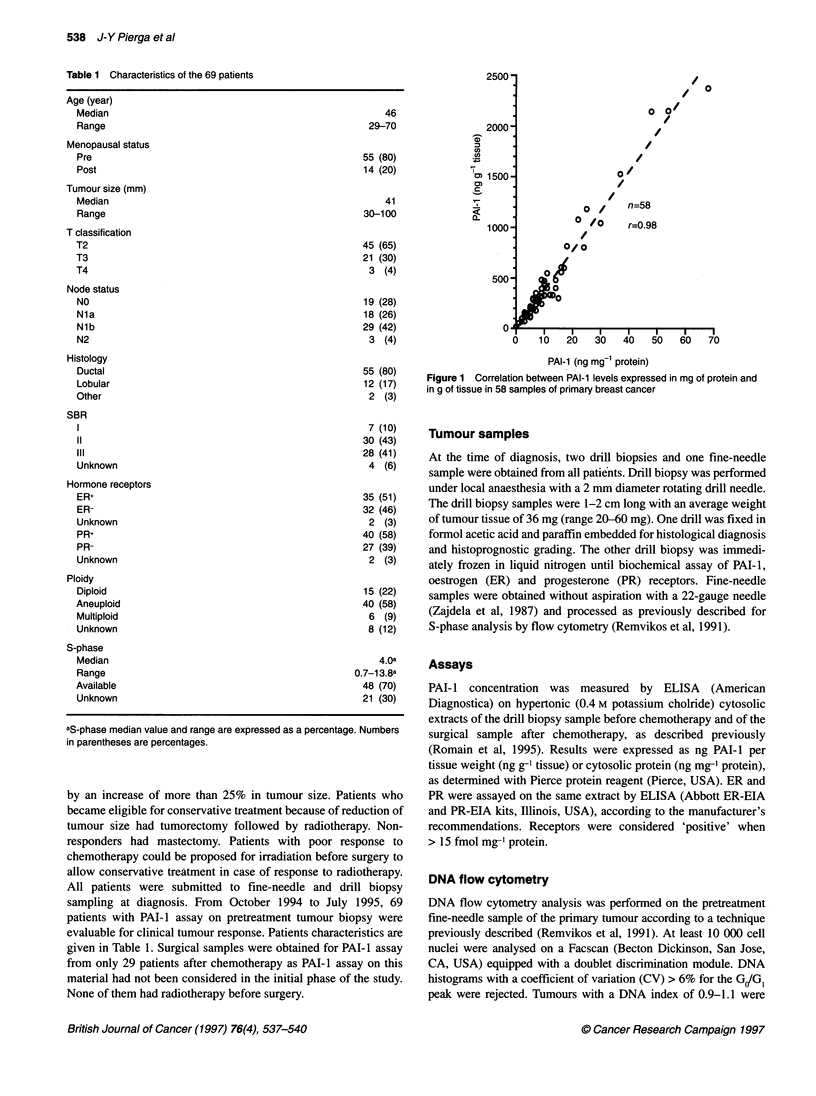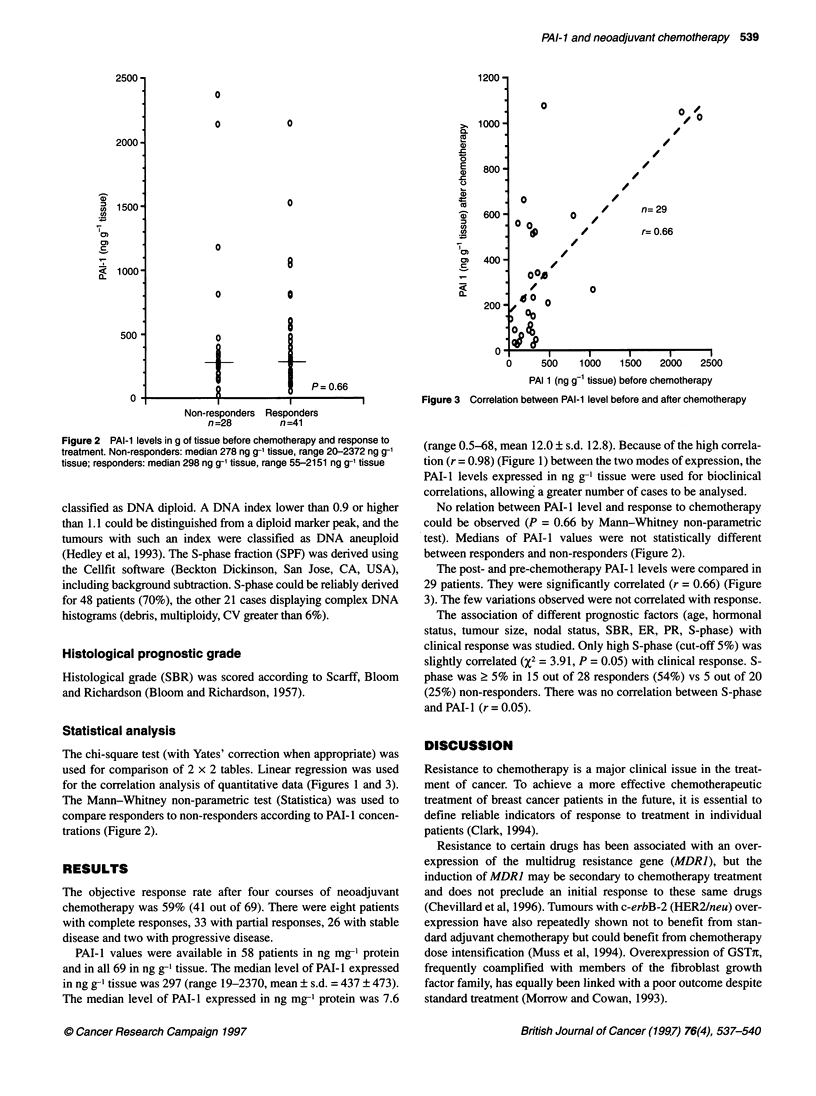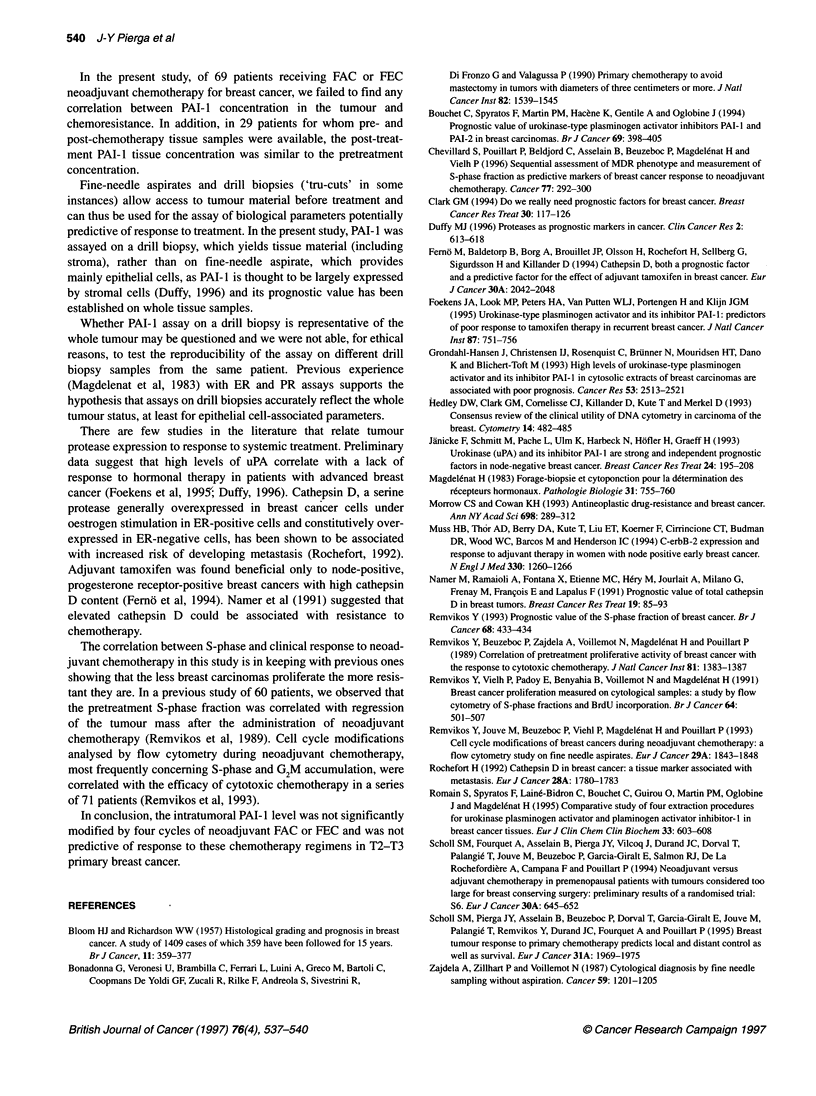# Plasminogen activator inhibitor-1 (PAI-1) is not related to response to neoadjuvant chemotherapy in breast cancer.

**DOI:** 10.1038/bjc.1997.421

**Published:** 1997

**Authors:** J. Y. Pierga, C. LainÃ©-Bidron, P. Beuzeboc, P. De CrÃ©moux, P. Pouillart, H. MagdelÃ©nat

**Affiliations:** Physiopathology Laboratory and Medical Oncology Department, Institute Curie, Paris, France.

## Abstract

There is no information available on the relation between response to chemotherapy and the high-risk phenotype assessed by uPA and/or PAI-1. The clinical situation of neoadjuvant chemotherapy provides a means of rapidly assessing the sensitivity of the primary tumour to cytotoxic drug regimens. The goal of the study was to assess prospectively the predictive value of PAI-1 for response to first-line chemotherapy. PAI-1 concentration was measured on hypertonic cytosolic extracts (0.4 M potassium chloride) by ELISA before chemotherapy on a drill biopsy sample of the tumour in 69 T2 and T3 breast cancer patients (median age 46 years). Oestrogen receptor (ER) (51% ER+), progesterone receptor (PR) (58% PR+), S-phase (median 4.0%) and ploidy were also assessed in the majority of cases. The clinical response to treatment was evaluated after four cycles of FAC or FEC regimen (5-fluorouracil, epidoxorubicin or doxorubicin and cyclophosphamide) (one cycle every 4th week). PAI-1 could be assayed in 29 post-chemotherapy surgical samples. The objective response rate (complete response plus partial response) was 59% (41 out of 69). PAI-1 expressed as gram of tissue (range 19-2370 ng g(-1) tissue) was highly correlated (r = 0.98) to PAI-1 expressed as mg protein (range 0.5-68 ng mg(-1) protein). No correlation between PAI-1 level and response could be observed, with any cut-off. The post- and pre-chemotherapy PAI-1 levels were correlated (r = 0.66). Of all biological parameters, only high S-phase (cut-off 5%) was slightly correlated (chi2 = 3.91, P = 0.05) to response. These data suggest that PAI-1 is not a predictive marker of response to chemotherapy in breast cancer and that its level is not altered by neoadjuvant chemotherapy.


British Journal of Cancer (1997) 76(4), 537–540
© 1997 Cancer Research Campaign

### Plasminogen activator inhibitor-1 (PAI-1) is not related to response to neoadjuvant chemotherapy in breast cancer

J-Y Pierga[1,2], C Lainé-Bidron[1], P Beuzeboc[2], P De Crémoux[1], P Pouillart[2] and H Magdelénat[1]

[1]Physiopathology Laboratory and [2]Medical Oncology Department, Institute Curie, 26, rue d'Ulm, 75231 Paris Cedex, France

**Summary** There is no information available on the relation between response to chemotherapy and the high-risk phenotype assessed by uPA and/or PAI-1. The clinical situation of neoadjuvant chemotherapy provides a means of rapidly assessing the sensitivity of the primary tumour to cytotoxic drug regimens. The goal of the study was to assess prospectively the predictive value of PAI-1 for response to first-line chemotherapy. PAI-1 concentration was measured on hypertonic cytosolic extracts (0.4 M potassium chloride) by ELISA before chemotherapy on a drill biopsy sample of the tumour in 69 T2 and T3 breast cancer patients (median age 46 years). Oestrogen receptor (ER) (51% ER+), progesterone receptor (PR) (58% PR+), S-phase (median 4.0%) and ploidy were also assessed in the majority of cases. The clinical response to treatment was evaluated after four cycles of FAC or FEC regimen (5-fluorouracil, epidoxorubicin or doxorubicin and cyclophosphamide) (one cycle every 4th week). PAI-1 could be assayed in 29 post-chemotherapy surgical samples. The objective response rate (complete response plus partial response) was 59% (41 out of 69). PAI-1 expressed as gram of tissue (range 19–2370 ng $g^{-1}$ tissue) was highly correlated ($r$ = 0.98) to PAI-1 expressed as mg protein (range 0.5–68 ng $mg^{-1}$ protein). No correlation between PAI-1 level and response could be observed, with any cut-off. The post- and pre-chemotherapy PAI-1 levels were correlated ($r$ = 0.66). Of all biological parameters, only high S-phase (cut-off 5%) was slightly correlated ($\chi^2$ = 3.91, $P$ = 0.05) to response. These data suggest that PAI-1 is not a predictive marker of response to chemotherapy in breast cancer and that its level is not altered by neoadjuvant chemotherapy.

**Keywords:** plasminogen activator inhibitor-1; breast cancer; neoadjuvant chemotherapy; S-phase fraction

Neoadjuvant chemotherapy provides a means of rapidly evaluating the sensitivity of a primary tumour to cytotoxic drug regimens. In breast cancer, neoadjuvant chemotherapy is used to improve breast preservation, although no survival advantage has yet been demonstrated (Scholl et al, 1994). It has been reported recently that breast tumour response to primary chemotherapy could predict local and distant control as well as survival (Scholl et al, 1995). Predictive tests of tumour response should be developed to more accurately select patients who may or may not benefit from such therapy (Bonadonna et al, 1990). In this respect, the measurement of S-phase fraction by flow cytometry (Remvikos et al, 1993) and detection of multidrug resistance (MDR) phenotype, before or during treatment, have recently emerged as promising predictive tests (Chevillard et al, 1996). It has been reported that the lysosomal protease cathepsin D might be associated with chemoresistance (Namer et al, 1991).

Urokinase plaminogen activator (uPA) is a proteolytic enzyme involved in processes leading to tumour invasion of surrounding tissues. Its activity during metastasis is regulated by an inhibitor, plasminogen activator inhibitor-1 (PAI-1). Previous studies have shown that high levels of uPA and PAI-1 are associated with poor prognosis in primary breast cancers (Grondahl-Hansen et al, 1993; Jänicke et al, 1993; Bouchet et al, 1994; Duffy et al, 1996). However, there is no information available on the relation between response to chemotherapy and the high-risk phenotype determined by uPA and/or PAI-1. Recently, Foekens et al (1995) have shown that the assay of uPA in primary breast tumours may be useful in predicting the overall response of metastatic disease to tamoxifen, patients with uPA-negative tumours exhibiting a better response.

The goal of this pilot study was to assess prospectively the predictive value of the expression levels of PAI-1 before first-line neoadjuvant chemotherapy and the response to this treatment in a series of 69 patients with primary operable breast cancer.

*Received 16 October 1996*
*Revised 27 February 1997*
*Accepted 4 March 1997*

*Correspondence to:* H Magdelénat

### MATERIAL AND METHODS

### Patients

Patients with operable tumours, 3 cm or more in size, with or without clinical node involvement, with a pathological diagnosis of invasive breast cancer established on a drill biopsy tumour sample and aged less than 70 years were eligible for neoadjuvant chemotherapy. Inflammatory, bilateral, locally advanced or metastatic breast cancer were exclusion criteria for this study. All patients received four cycles of neoadjuvant FEC or FAC chemotherapy regimen (5-fluorouracil, epidoxorubicin or doxorubicin and cyclophosphamide) before locoregional treatment. Chemotherapy courses were administered every 4 weeks. Tumour response was evaluated clinically and radiologically after 4 months of treatment. Responders were defined by either a complete response (CR, disappearance of clinically palpable disease) or partial response (PR, reduction of more than 50% of the product of the two largest tumour diameters). Stable disease was defined by no change in tumour size and progressive disease

**Table 1** Characteristics of the 69 patients

| | |
|---|---|
| Age (year) | |
| Median | 46 |
| Range | 29–70 |
| Menopausal status | |
| Pre | 55 (80) |
| Post | 14 (20) |
| Tumour size (mm) | |
| Median | 41 |
| Range | 30–100 |
| T classification | |
| T2 | 45 (65) |
| T3 | 21 (30) |
| T4 | 3 (4) |
| Node status | |
| N0 | 19 (28) |
| N1a | 18 (26) |
| N1b | 29 (42) |
| N2 | 3 (4) |
| Histology | |
| Ductal | 55 (80) |
| Lobular | 12 (17) |
| Other | 2 (3) |
| SBR | |
| I | 7 (10) |
| II | 30 (43) |
| III | 28 (41) |
| Unknown | 4 (6) |
| Hormone receptors | |
| $ER^+$ | 35 (51) |
| $ER^-$ | 32 (46) |
| Unknown | 2 (3) |
| $PR^+$ | 40 (58) |
| $PR^-$ | 27 (39) |
| Unknown | 2 (3) |
| Ploidy | |
| Diploid | 15 (22) |
| Aneuploid | 40 (58) |
| Multiploid | 6 (9) |
| Unknown | 8 (12) |
| S-phase | |
| Median | 4.0[a] |
| Range | 0.7–13.8[a] |
| Available | 48 (70) |
| Unknown | 21 (30) |

[a]S-phase median value and range are expressed as a percentage. Numbers in parentheses are percentages.

by an increase of more than 25% in tumour size. Patients who became eligible for conservative treatment because of reduction of tumour size had tumorectomy followed by radiotherapy. Non-responders had mastectomy. Patients with poor response to chemotherapy could be proposed for irradiation before surgery to allow conservative treatment in case of response to radiotherapy. All patients were submitted to fine-needle and drill biopsy sampling at diagnosis. From October 1994 to July 1995, 69 patients with PAI-1 assay on pretreatment tumour biopsy were evaluable for clinical tumour response. Patients characteristics are given in Table 1. Surgical samples were obtained for PAI-1 assay from only 29 patients after chemotherapy as PAI-1 assay on this material had not been considered in the initial phase of the study. None of them had radiotherapy before surgery.

**Figure 1** Correlation between PAI-1 levels expressed in mg of protein and in g of tissue in 58 samples of primary breast cancer

### Tumour samples

At the time of diagnosis, two drill biopsies and one fine-needle sample were obtained from all patients. Drill biopsy was performed under local anaesthesia with a 2 mm diameter rotating drill needle. The drill biopsy samples were 1–2 cm long with an average weight of tumour tissue of 36 mg (range 20–60 mg). One drill was fixed in formol acetic acid and paraffin embedded for histological diagnosis and histoprognostic grading. The other drill biopsy was immediately frozen in liquid nitrogen until biochemical assay of PAI-1, oestrogen (ER) and progesterone (PR) receptors. Fine-needle samples were obtained without aspiration with a 22-gauge needle (Zajdela et al, 1987) and processed as previously described for S-phase analysis by flow cytometry (Remvikos et al, 1991).

### Assays

PAI-1 concentration was measured by ELISA (American Diagnostica) on hypertonic (0.4 M potassium cholride) cytosolic extracts of the drill biopsy sample before chemotherapy and of the surgical sample after chemotherapy, as described previously (Romain et al, 1995). Results were expressed as ng PAI-1 per tissue weight (ng $g^{-1}$ tissue) or cytosolic protein (ng $mg^{-1}$ protein), as determined with Pierce protein reagent (Pierce, USA). ER and PR were assayed on the same extract by ELISA (Abbott ER-EIA and PR-EIA kits, Illinois, USA), according to the manufacturer's recommendations. Receptors were considered 'positive' when > 15 fmol $mg^{-1}$ protein.

### DNA flow cytometry

DNA flow cytometry analysis was performed on the pretreatment fine-needle sample of the primary tumour according to a technique previously described (Remvikos et al, 1991). At least 10 000 cell nuclei were analysed on a Facscan (Becton Dickinson, San Jose, CA, USA) equipped with a doublet discrimination module. DNA histograms with a coefficient of variation (CV) > 6% for the $G_0/G_1$ peak were rejected. Tumours with a DNA index of 0.9–1.1 were

 © Cancer Research Campaign 1997

**Figure 2** PAI-1 levels in g of tissue before chemotherapy and response to treatment. Non-responders: median 278 ng $g^{-1}$ tissue, range 20–2372 ng $g^{-1}$ tissue; responders: median 298 ng $g^{-1}$ tissue, range 55–2151 ng $g^{-1}$ tissue

**Figure 3** Correlation between PAI-1 level before and after chemotherapy classified as DNA diploid. A DNA index lower than 0.9 or higher than 1.1 could be distinguished from a diploid marker peak, and the tumours with such an index were classified as DNA aneuploid (Hedley et al, 1993). The S-phase fraction (SPF) was derived using the Cellfit software (Beckton Dickinson, San Jose, CA, USA), including background subtraction. S-phase could be reliably derived for 48 patients (70%), the other 21 cases displaying complex DNA histograms (debris, multiploidy, CV greater than 6%).

### Histological prognostic grade

Histological grade (SBR) was scored according to Scarff, Bloom and Richardson (Bloom and Richardson, 1957).

### Statistical analysis

The chi-square test (with Yates' correction when appropriate) was used for comparison of 2 × 2 tables. Linear regression was used for the correlation analysis of quantitative data (Figures 1 and 3). The Mann–Whitney non-parametric test (Statistica) was used to compare responders to non-responders according to PAI-1 concentrations (Figure 2).

### RESULTS

The objective response rate after four courses of neoadjuvant chemotherapy was 59% (41 out of 69). There were eight patients with complete responses, 33 with partial responses, 26 with stable disease and two with progressive disease.

PAI-1 values were available in 58 patients in ng $mg^{-1}$ protein and in all 69 in ng $g^{-1}$ tissue. The median level of PAI-1 expressed in ng $g^{-1}$ tissue was 297 (range 19–2370, mean ± s.d. = 437 ± 473). The median level of PAI-1 expressed in ng $mg^{-1}$ protein was 7.6 (range 0.5–68, mean 12.0 ± s.d. 12.8). Because of the high correlation ($r = 0.98$) (Figure 1) between the two modes of expression, the PAI-1 levels expressed in ng $g^{-1}$ tissue were used for bioclinical correlations, allowing a greater number of cases to be analysed.

No relation between PAI-1 level and response to chemotherapy could be observed ($P = 0.66$ by Mann–Whitney non-parametric test). Medians of PAI-1 values were not statistically different between responders and non-responders (Figure 2).

The post- and pre-chemotherapy PAI-1 levels were compared in 29 patients. They were significantly correlated ($r = 0.66$) (Figure 3). The few variations observed were not correlated with response.

The association of different prognostic factors (age, hormonal status, tumour size, nodal status, SBR, ER, PR, S-phase) with clinical response was studied. Only high S-phase (cut-off 5%) was slightly correlated ($\chi^2 = 3.91$, $P = 0.05$) with clinical response. S-phase was ≥ 5% in 15 out of 28 responders (54%) vs 5 out of 20 (25%) non-responders. There was no correlation between S-phase and PAI-1 ($r = 0.05$).

### DISCUSSION

Resistance to chemotherapy is a major clinical issue in the treatment of cancer. To achieve a more effective chemotherapeutic treatment of breast cancer patients in the future, it is essential to define reliable indicators of response to treatment in individual patients (Clark, 1994).

Resistance to certain drugs has been associated with an overexpression of the multidrug resistance gene (*MDR1*), but the induction of *MDR1* may be secondary to chemotherapy treatment and does not preclude an initial response to these same drugs (Chevillard et al, 1996). Tumours with c-*erb*B-2 (HER2/*neu*) overexpression have also repeatedly shown not to benefit from standard adjuvant chemotherapy but could benefit from chemotherapy dose intensification (Muss et al, 1994). Overexpression of GSTπ, frequently coamplified with members of the fibroblast growth factor family, has equally been linked with a poor outcome despite standard treatment (Morrow and Cowan, 1993).

© Cancer Research Campaign 1997

In the present study, of 69 patients receiving FAC or FEC neoadjuvant chemotherapy for breast cancer, we failed to find any correlation between PAI-1 concentration in the tumour and chemoresistance. In addition, in 29 patients for whom pre- and post-chemotherapy tissue samples were available, the post-treatment PAI-1 tissue concentration was similar to the pretreatment concentration.

Fine-needle aspirates and drill biopsies ('tru-cuts' in some instances) allow access to tumour material before treatment and can thus be used for the assay of biological parameters potentially predictive of response to treatment. In the present study, PAI-1 was assayed on a drill biopsy, which yields tissue material (including stroma), rather than on fine-needle aspirate, which provides mainly epithelial cells, as PAI-1 is thought to be largely expressed by stromal cells (Duffy, 1996) and its prognostic value has been established on whole tissue samples.

Whether PAI-1 assay on a drill biopsy is representative of the whole tumour may be questioned and we were not able, for ethical reasons, to test the reproducibility of the assay on different drill biopsy samples from the same patient. Previous experience (Magdelenat et al, 1983) with ER and PR assays supports the hypothesis that assays on drill biopsies accurately reflect the whole tumour status, at least for epithelial cell-associated parameters.

There are few studies in the literature that relate tumour protease expression to response to systemic treatment. Preliminary data suggest that high levels of uPA correlate with a lack of response to hormonal therapy in patients with advanced breast cancer (Foekens et al, 1995; Duffy, 1996). Cathepsin D, a serine protease generally overexpressed in breast cancer cells under oestrogen stimulation in ER-positive cells and constitutively over-expressed in ER-negative cells, has been shown to be associated with increased risk of developing metastasis (Rochefort, 1992). Adjuvant tamoxifen was found beneficial only to node-positive, progesterone receptor-positive breast cancers with high cathepsin D content (Fernö et al, 1994). Namer et al (1991) suggested that elevated cathepsin D could be associated with resistance to chemotherapy.

The correlation between S-phase and clinical response to neoadjuvant chemotherapy in this study is in keeping with previous ones showing that the less breast carcinomas proliferate the more resistant they are. In a previous study of 60 patients, we observed that the pretreatment S-phase fraction was correlated with regression of the tumour mass after the administration of neoadjuvant chemotherapy (Remvikos et al, 1989). Cell cycle modifications analysed by flow cytometry during neoadjuvant chemotherapy, most frequently concerning S-phase and $G_2M$ accumulation, were correlated with the efficacy of cytotoxic chemotherapy in a series of 71 patients (Remvikos et al, 1993).

In conclusion, the intratumoral PAI-1 level was not significantly modified by four cycles of neoadjuvant FAC or FEC and was not predictive of response to these chemotherapy regimens in T2–T3 primary breast cancer.

### REFERENCES

Bloom HJ and Richardson WW (1957) Histological grading and prognosis in breast cancer. A study of 1409 cases of which 359 have been followed for 15 years. *Br J Cancer*, **11**: 359–377

Bonadonna G, Veronesi U, Brambilla C, Ferrari L, Luini A, Greco M, Bartoli C, Coopmans De Yoldi GF, Zucali R, Rilke F, Andreola S, Sivestrini R, Di Fronzo G and Valagussa P (1990) Primary chemotherapy to avoid mastectomy in tumors with diameters of three centimeters or more. *J Natl Cancer Inst* **82**: 1539–1545

Bouchet C, Spyratos F, Martin PM, Hacène K, Gentile A and Oglobine J (1994) Prognostic value of urokinase-type plasminogen activator inhibitors PAI-1 and PAI-2 in breast carcinomas. *Br J Cancer* **69**: 398–405

Chevillard S, Pouillart P, Beldjord C, Asselain B, Beuzeboc P, Magdelénat H and Vielh P (1996) Sequential assessment of MDR phenotype and measurement of S-phase fraction as predictive markers of breast cancer response to neoadjuvant chemotherapy. *Cancer* **77**: 292–300

Clark GM (1994) Do we really need prognostic factors for breast cancer. *Breast Cancer Res Treat* **30**: 117–126

Duffy MJ (1996) Proteases as prognostic markers in cancer. *Clin Cancer Res* **2**: 613–618

Fernö M, Baldetorp B, Borg A, Brouillet JP, Olsson H, Rochefort H, Sellberg G, Sigurdsson H and Killander D (1994) Cathepsin D, both a prognostic factor and a predictive factor for the effect of adjuvant tamoxifen in breast cancer. *Eur J Cancer* **30A**: 2042–2048

Foekens JA, Look MP, Peters HA, Van Putten WLJ, Portengen H and Klijn JGM (1995) Urokinase-type plasminogen activator and its inhibitor PAI-1: predictors of poor response to tamoxifen therapy in recurrent breast cancer. *J Natl Cancer Inst* **87**: 751–756

Grondahl-Hansen J, Christensen IJ, Rosenquist C, Brünner N, Mouridsen HT, Dano K and Blichert-Toft M (1993) High levels of urokinase-type plasminogen activator and its inhibitor PAI-1 in cytosolic extracts of breast carcinomas are associated with poor prognosis. *Cancer Res* **53**: 2513–2521

Hedley DW, Clark GM, Cornelisse CJ, Killander D, Kute T and Merkel D (1993) Consensus review of the clinical utility of DNA cytometry in carcinoma of the breast. *Cytometry* **14**: 482–485

Jänicke F, Schmitt M, Pache L, Ulm K, Harbeck N, Höfler H, Graeff H (1993) Urokinase (uPA) and its inhibitor PAI-1 are strong and independent prognostic factors in node-negative breast cancer. *Breast Cancer Res Treat* **24**: 195–208

Magdelénat H (1983) Forage-biopsie et cytoponction pour la détermination des récepteurs hormonaux. *Pathologie Biologie* **31**: 755–760

Morrow CS and Cowan KH (1993) Antineoplastic drug-resistance and breast cancer. *Ann NY Acad Sci* **698**: 289–312

Muss HB, Thor AD, Berry DA, Kute T, Liu ET, Koerner F, Cirrincione CT, Budman DR, Wood WC, Barcos M and Henderson IC (1994) C-erbB-2 expression and response to adjuvant therapy in women with node positive early breast cancer. *N Engl J Med* **330**: 1260–1266

Namer M, Ramaioli A, Fontana X, Etienne MC, Héry M, Jourlait A, Milano G, Frenay M, François E and Lapalus F (1991) Prognostic value of total cathepsin D in breast tumors. *Breast Cancer Res Treat* **19**: 85–93

Remvikos Y (1993) Prognostic value of the S-phase fraction of breast cancer. *Br J Cancer* **68**: 433–434

Remvikos Y, Beuzeboc P, Zajdela A, Voillemot N, Magdelénat H and Pouillart P (1989) Correlation of pretreatment proliferative activity of breast cancer with the response to cytotoxic chemotherapy. *J Natl Cancer Inst* **81**: 1383–1387

Remvikos Y, Vielh P, Padoy E, Benyahia B, Voillemot N and Magdelénat H (1991) Breast cancer proliferation measured on cytological samples: a study by flow cytometry of S-phase fractions and BrdU incorporation. *Br J Cancer* **64**: 501–507

Remvikos Y, Jouve M, Beuzeboc P, Viehl P, Magdelénat H and Pouillart P (1993) Cell cycle modifications of breast cancers during neoadjuvant chemotherapy: a flow cytometry study on fine needle aspirates. *Eur J Cancer* **29A**: 1843–1848

Rochefort H (1992) Cathepsin D in breast cancer: a tissue marker associated with metastasis. *Eur J Cancer* **28A**: 1780–1783

Romain S, Spyratos F, Lainé-Bidron C, Bouchet C, Guirou O, Martin PM, Oglobine J and Magdelénat H (1995) Comparative study of four extraction procedures for urokinase plasminogen activator and plaminogen activator inhibitor-1 in breast cancer tissues. *Eur J Clin Chem Clin Biochem* **33**: 603–608

Scholl SM, Fourquet A, Asselain B, Pierga JY, Vilcoq J, Durand JC, Dorval T, Palangié T, Jouve M, Beuzeboc P, Garcia-Giralt E, Salmon RJ, De La Rochefordière A, Campana F and Pouillart P (1994) Neoadjuvant versus adjuvant chemotherapy in premenopausal patients with tumours considered too large for breast conserving surgery: preliminary results of a randomised trial: S6. *Eur J Cancer* **30A**: 645–652

Scholl SM, Pierga JY, Asselain B, Beuzeboc P, Dorval T, Garcia-Giralt E, Jouve M, Palangié T, Remvikos Y, Durand JC, Fourquet A and Pouillart P (1995) Breast tumour response to primary chemotherapy predicts local and distant control as well as survival. *Eur J Cancer* **31A**: 1969–1975

Zajdela A, Zillhart P and Voillemot N (1987) Cytological diagnosis by fine needle sampling without aspiration. *Cancer* **59**: 1201–1205

© Cancer Research Campaign 1997